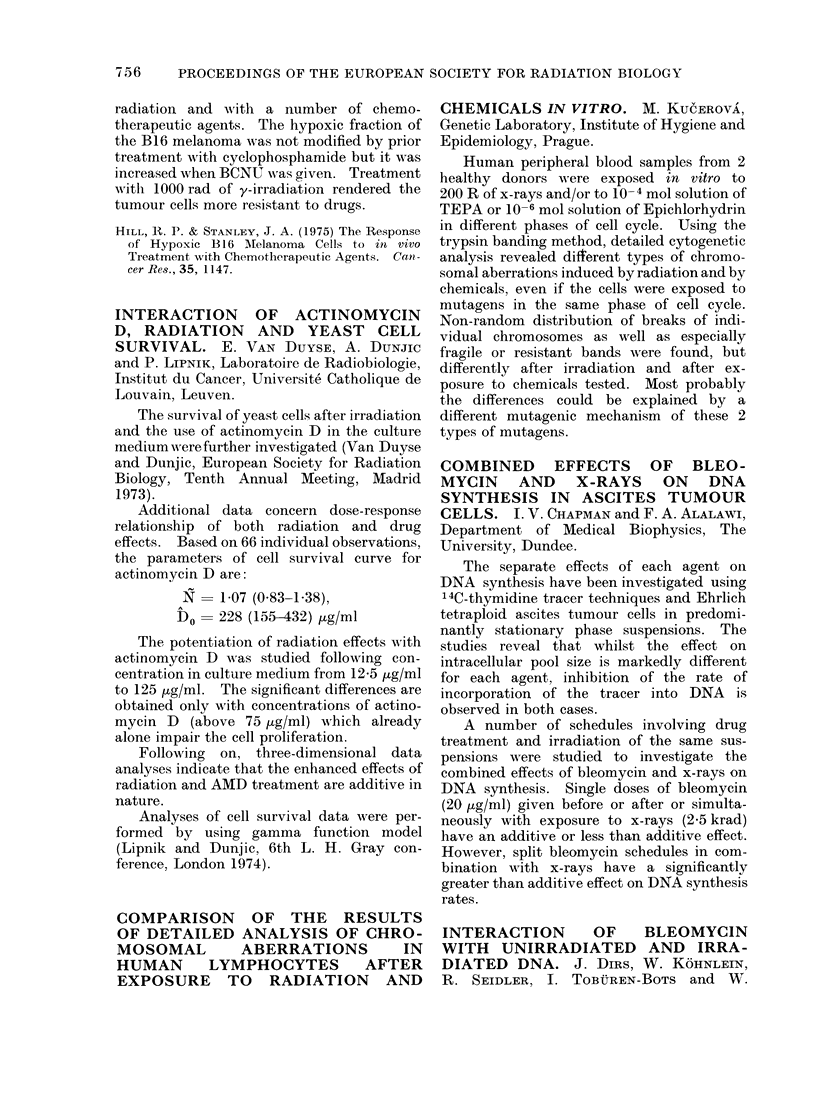# Proceedings: Interaction of actinomycin D, radiation and yeast cell survival.

**DOI:** 10.1038/bjc.1975.304

**Published:** 1975-12

**Authors:** E. van Duyse, A. Dunjic, P. Lipnik


### INTERACTION OF ACTINOMYCIN D, RADIATION AND YEAST CELL SURVIVAL.

E. VAN DUYSE, A. DUNJIC and P. LIPNIK, Laboratoire de Radiobiologie, Institut du Cancer, Université Catholique de Louvain, Leuven.

The survival of yeast cells after irradiation and the use of actinomycin D in the culture medium were further investigated (Van Duyse and Dunjic, European Society for Radiation Biology, Tenth Annual Meeting, Madrid 1973).

Additional data concern dose-response relationship of both radiation and drug effects. Based on 66 individual observations, the parameters of cell survival curve for actinomycin D are:

$$\tilde{N} = 1{\cdot}07\ (0{\cdot}83\text{–}1{\cdot}38),$$

$$\hat{D}_0 = 228\ (155\text{–}432)\ \mu g/ml$$

The potentiation of radiation effects with actinomycin D was studied following concentration in culture medium from 12·5 $\mu$g/ml to 125 $\mu$g/ml. The significant differences are obtained only with concentrations of actinomycin D (above 75 $\mu$g/ml) which already alone impair the cell proliferation.

Following on, three-dimensional data analyses indicate that the enhanced effects of radiation and AMD treatment are additive in nature.

Analyses of cell survival data were performed by using gamma function model (Lipnik and Dunjic, 6th L. H. Gray conference, London 1974).